# Construction of a container isolation ward: A rapidly scalable modular approach to expand isolation capacity during the coronavirus disease 2019 (COVID-19) pandemic

**DOI:** 10.1017/ice.2020.1222

**Published:** 2020-09-23

**Authors:** Liang En Wee, Esther Monica Peijin Fan, Raphael Heng, Shin Yuh Ang, Juat Lan Chiang, Thuan Tong Tan, Moi Lin Ling, Limin Wijaya

**Affiliations:** 1 SingHealth Infectious Diseases Residency, Singapore; 2 Department of Infectious Diseases, Singapore General Hospital, Singapore; 3 Division of Nursing, Singapore General Hospital, Singapore; 4 Facilities and Infrastructure Development, Singapore General Hospital, Singapore; 5 Department of Infection Prevention and Epidemiology, Singapore General Hospital, Singapore

*To the Editor*—During the ongoing coronavirus disease 2019 (COVID-19) pandemic, airborne infection isolation rooms (AIIRs) are in high demand. Surge capacity is urgently required during significant ongoing community transmission.^1^ Converting hospital wards to AIIRs can serve as a temporary solution,^1,2^ but it comes at the expense of existing capacity. Temporary tent-based structures or conversion of nonmedical facilities has been commonly proposed.^3-5^ Such solutions, though, may not be durable, and retrofitting nonmedical facilities to meet medical standards is difficult. Building more permanent structures with AIIR capabilities, however, is time-consuming and costly.^5^ We describe our institution’s experience with constructing an isolation ward from prefabricated containers.

In Singapore, at the end of July 2020, >50,000 cases of COVID-19 had been reported locally.^6^ Our institution’s purpose-built 51-bed isolation ward housed high-risk COVID-19 suspects and confirmed COVID-19 cases in AIIRs. From February 2020, given ongoing community transmission, lower-risk individuals with respiratory syndromes were housed in converted general wards, with partitions and reduced beds per cubicle to mitigate the risk of healthcare-associated transmission.^7^ To date, this containment strategy has been successful, with no cases of healthcare-associated transmission between patients and healthcare workers (HCWs).^8,9^ However, this placed pressure on bed capacity, with almost 20% of bed capacity set aside to house suspected and confirmed cases of COVID-19. Our institution thus sought to relieve the pressure by constructing an isolation ward extension.

**Fig. 1.**
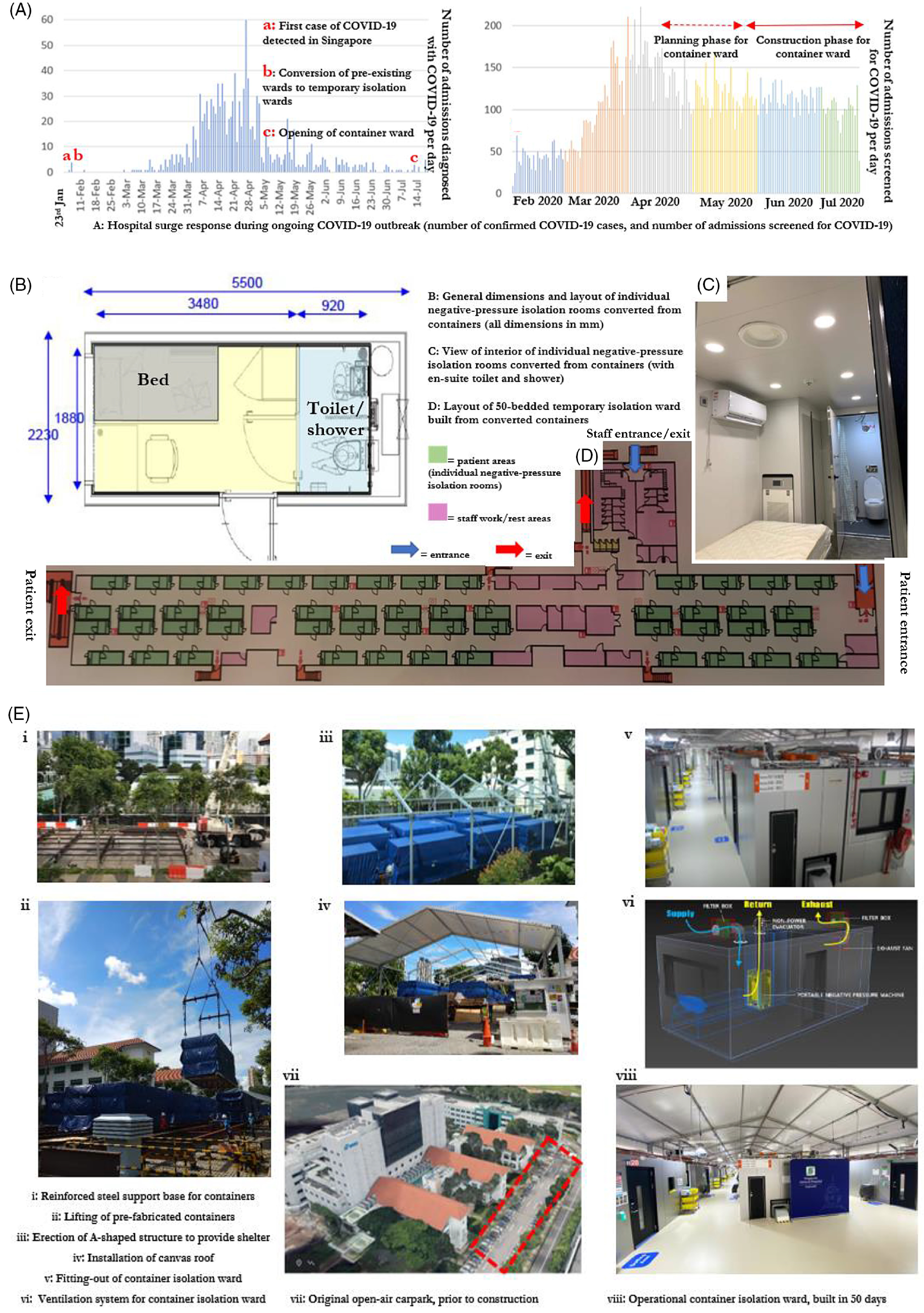
Hospital surge response over 6 months during COVID-19 outbreak, layout of container isolation ward, and construction process.

From mid-April 2020, our institution started planning the isolation-ward extension, soon after a surge in cases (Fig. 1a). An open-air car park (3,200 m^2^) was utilized. The novel feature was the utilization of a scalable modular design using prefabricated containers. Each prefabricated container (6 m × 2.4 m) was redesigned as a single-occupancy room with an en suite bathroom (Fig. 1b, c, and d). All patient rooms met design standards for AIIRs, including ≥12 air changes per hour and controlled direction of air flow with a negative differential pressure of −2.5 Pascal. Each container was also equipped with oxygen, air conditioning, exhaust ventilation, and a high-efficiency particulate air (HEPA) filter. Modification of containers was done off site. One container was also customized with lead shielding and a patient booth to allow x-rays to be performed.

At the end of May 2020, construction began (Fig. 1e). Modular construction allowed rapid construction of a 50-bed isolation ward in just 50 days, using only 100 workers, despite significant challenges. Outbreaks in the dormitories of foreign workers significantly limited manpower availability, and construction work was curtailed during a 2-month-long shutdown as part of community-based measures to reduce transmission.^6^ During construction, the prefabricated containers were placed on a reinforced base. Over the containers, an A-shaped structure with a canvas roof was erected to provide shelter. Nurses’ stations, a rest area, changing rooms, and on-call rooms were also constructed within the structure. The layout incorporated segregated, unidirectional traffic flows for entry and exit of HCWs and patients to reduce the possibility of cross contamination. Water, electricity, sewage, and oxygen were connected to the hospital’s utilities system.

The container isolation ward opened on July 14, 2020. Staff don N95 respirators when working within the ward, and full PPE (N95 respirator, disposable gown, gloves and eye protection) during patient contact. The ward’s capacity is equally divided between confirmed COVID-19 cases and high-risk COVID-19 suspects. Operations have been designed to minimize potential exposure. For monitoring vitals, patients are given wearable biosensors that wirelessly transmit heart rate, respiration rate, and oxygen saturation to a mobile app, allowing continuous remote monitoring. In-room smart phones are also provided; built-in video-conferencing allows for medical, nursing and pharmacy staff to communicate remotely with the patient. Nursing and medical manpower has been drawn from various hospital departments. Training has been provided for all HCWs. For nurses who did not have previous experience in units nursing COVID-19 suspects or COVID-19 cases, they had to undergo a few weeks of attachment in the isolation units to be trained in strict infection prevention and control (IPC) practices. To date, no IPC breaches or exposures have been observed over 1 month of operations, despite regular audit. Overall, >1,500 cases of COVID-19 have been managed at our institution.

Certain limitations were anticipated. Observation was limited because each container only had a single window. As such, the usage of wearable biosensors required incorporation of network equipment and wireless access points during the design phase. Given the limited space, it was anticipated that the psychological effects of isolation might pose difficulties.^10^ Provision of an in-room smart phone served as a communication conduit, improving the experience of isolation. Limited space meant that resuscitation would be challenging. As such, admission to the container isolation ward was restricted to patients <75 years of age who were functionally independent and had little risk of immediate clinical deterioration. A fully equipped resuscitation room was built within the structure to accommodate resuscitation in full PPE in the event of collapse. However, to minimize the likelihood of activation, deteriorating patients would be pre-emptively transferred to the main hospital block.

In conclusion, prefabricated containers allowed rapid expansion of AIIR capacity using an easily scalable modular design, though space constraints meant that patient selection had to be optimized and patient monitoring facilitated. This flexible modular approach provides surge capacity for isolation facilities, preventing hospitals from being overwhelmed during a pandemic caused by a novel respiratory pathogen. Other cases requiring airborne precautions, such as pulmonary tuberculosis and varicella, have also been managed in the container isolation ward; the container isolation ward is thus anticipated to provide AIIR capacity even after the height of the COVID-19 pandemic is over.
